# Emerging antioxidant therapies in Friedreich’s ataxia

**DOI:** 10.3389/fphar.2024.1359618

**Published:** 2024-02-06

**Authors:** Fred Jonathan Edzeamey, Zenouska Ramchunder, Charareh Pourzand, Sara Anjomani Virmouni

**Affiliations:** ^1^ Ataxia Research Group, Division of Biosciences, Department of Life Sciences, College of Health, Medicine, and Life Sciences (CHMLS), Brunel University London, Uxbridge, United Kingdom; ^2^ Department of Life Sciences, University of Bath, Bath, United Kingdom; ^3^ Centre for Therapeutic Innovation, University of Bath, Bath, United Kingdom

**Keywords:** Friedreich’s ataxia, antioxidants, clinical trials, oxidative stress, reactive oxygen species

## Abstract

Friedreich’s ataxia (FRDA) is a rare childhood neurologic disorder, affecting 1 in 50,000 Caucasians. The disease is caused by the abnormal expansion of the GAA repeat sequence in intron 1 of the *FXN* gene, leading to the reduced expression of the mitochondrial protein frataxin. The disease is characterised by progressive neurodegeneration, hypertrophic cardiomyopathy, diabetes mellitus and musculoskeletal deformities. The reduced expression of frataxin has been suggested to result in the downregulation of endogenous antioxidant defence mechanisms and mitochondrial bioenergetics, and the increase in mitochondrial iron accumulation thereby leading to oxidative stress. The confirmation of oxidative stress as one of the pathological signatures of FRDA led to the search for antioxidants which can be used as therapeutic modality. Based on this observation, antioxidants with different mechanisms of action have been explored for FRDA therapy since the last two decades. In this review, we bring forth all antioxidants which have been investigated for FRDA therapy and have been signed off for clinical trials. We summarise their various target points in FRDA disease pathway, their performances during clinical trials and possible factors which might have accounted for their failure or otherwise during clinical trials. We also discuss the limitation of the studies completed and propose possible strategies for combinatorial therapy of antioxidants to generate synergistic effect in FRDA patients.

## 1 Introduction

Friedreich’s ataxia (FRDA) is a rare lethal autosomal recessive (Li et al., 2022) neurodegenerative disorder affecting 1 in 50,000 Caucasians (Vankan, 2013). The disease affects the brain, spinal cord, heart, muscles, and beta (β) cells of the pancreas, thereby characterising the disease by progressive neurodegeneration, musculoskeletal deformities, diabetes mellitus and hypertrophic cardiomyopathy (Anjomani Virmouni et al., 2014; Garg et al., 2017). The disease as of now has no cure. However, in February 2023, Reata Pharmaceuticals announced that the antioxidant Omaveloxolone has been approved by the US Food and Drug Administration (FDA) as the first treatment modality for FRDA (Lee, 2023). FRDA is caused by the abnormal expansion of the Guanine-Adenine-Adenine (GAA) repeat sequence in intron 1 of the *FXN* gene (Campuzano et al., 1996; Anjomani Virmouni et al., 2015). The *FXN* gene codes for a very important mitochondrial protein called frataxin (Cotticelli et al., 2022; Huichalaf et al., 2022). Frataxin is highly conserved in nature with homologs in mammals, plants, yeast and invertebrates. This 14 KDa protein is synthesised in the cytoplasm and, following cleavage by mitochondrial processing peptidase (MPP) to obtain the mature frataxin, is transported to the mitochondrial matrix (Acquaviva et al., 2008; Castaldo et al., 2008; Coppola et al., 2009; Marmolino and Acquaviva, 2009; Marmolino et al., 2010; Weng et al., 2020). Although the exact functions of this mitochondrial protein have not been well established, the downregulation of frataxin in various cell systems has led to some suggested functions for frataxin. It has been suggested that frataxin is involved in the synthesis of iron-sulfur clusters, metabolism of heme, regulation of oxidative phosphorylation and mitochondrial bioenergetics amongst others (Moreno-Cermeño et al., 2010; Bayot and Rustin, 2013; Anjomani Virmouni et al., 2015; Jasoliya et al., 2017; Alsina et al., 2018). Therefore, the reduced expression of the *FXN* gene and its protein frataxin, as observed in FRDA patients, has been associated with mitochondrial iron overload, reduced ATP production, oxidative stress, downregulation of antioxidant response element (ARE) -regulated genes and consequential cell death of sensory neurons in the dorsal root ganglia (DRG) and dentate nucleus (Nguyen et al., 2003; Moreno-Cermeño et al., 2010; Bayot and Rustin, 2013; Anjomani Virmouni et al., 2015; Jasoliya et al., 2017; Alsina et al., 2018).

## 2 Clinicopathologic characteristics of FRDA

Based on the onset of the disease, the disease can be classified into three categories, namely the classical FRDA (when the symptoms are present between the ages of 10 and 16 years), late onset FRDA (LOFA) (when the symptoms are present after the age of 25), and very late onset FRDA (VLOFA) (where the symptoms are present after the age of 40). FRDA is characterised by multisystem pathology, and this poses an essential threat to the health of patients with FRDA. The disease affects the central and peripheral nervous systems, the endocrine system (pancreas), the myocardium, and the musculoskeletal system (Cook and Giunti, 2017). The phenotype commonly associated with the classical FRDA includes dysarthria, gait and limb ataxia, and loss of lower limb reflexes accompanied by deep sensory loss (Morral et al., 2010). The loss of balance accompanied by trunk ataxia leads to the demand for patients to be wheelchair-bound by the third decade of the disease (Koeppen and Mazurkiewicz, 2013). The efficiency in performing basic daily activities becomes extremely difficult as the limb ataxia creates serious coordination deficits (Koeppen and Mazurkiewicz, 2013). Some of the early common signs include nose-finger ataxia, impaired heel-shin slide, and upper limb dysdiadochokinesia (Koeppen, 2011). Spasticity and pyramidal weakness of the lower limbs are associated with the advanced and late stages of the disease, respectively (Koeppen, 2011). Although spasticity has been associated with the advanced stages of the disease, this has also been observed in some ambulant patients and in those whose disease durations were less than 10 years (Milne et al., 2016). *Scoliosis*, *talipes equinovarus* and *pes cavus* are the notable musculoskeletal abnormalities associated with FRDA (Milne et al., 2016). The neurological phenotype of FRDA includes cerebellar atrophy in the dentate nucleus, the dorsal root ganglia become smaller, reduction in the spinal cord diameter, and loss of corticospinal and spinocerebellar tracts (Koeppen, 2011; Morral et al., 2010). Cardiomyopathy, the major cause of death amongst FRDA patients, and diabetes mellitus are also highly associated with FRDA patients (Kipps et al., 2009; Garg et al., 2017).

### 2.1 Ataxia rating scales

The severity of the ataxia symptoms displayed by those suffering from FRDA, as well as the efficacy of therapeutics in clinical trials for treatment of the disease, can be measured using a variety of different ataxia rating scales. Scales which measure ataxia in a more general sense include the International Cooperative Ataxia Rating Scale (ICARS) (Trouillas et al., 1997) and the Scale of the Assessment and Rating of Ataxia (SARA) (Schmitz-Hübsch et al., 2006; Grobe-Einsler et al., 2023). The ICARS, designed in 1997, evaluates four major aspects of ataxia: posture and stance, limb movement, speech disorders, and oculomotor disorders (Trouillas et al., 1997). Each aspect is measured using various tests, quantitatively rated, and added together to achieve a final rating out of 100 (Trouillas et al., 1997). The SARA, designed in 2004, is a simpler method of measurement, evaluates eight major aspects of ataxia: gait, stance, sitting, speech disturbance, finger-chase, nose-finger test, fast alternating hand movements, and heel-shin slide (Schmitz-Hübsch et al., 2006; Grobe-Einsler et al., 2023). The total SARA score is rated out of 40 (Schmitz-Hübsch et al., 2006; Grobe-Einsler et al., 2023).

In addition to these more generalised ataxia scales, there are also scales which focus more on measuring the symptoms specifically associated with FRDA. These include the Friedreich’s Ataxia Rating Scale (FARS) and the modified Friedreich’s Ataxia Rating Scale (mFARS) (FARA, 2023a). The FARS was introduced in 2005 and evaluates bulbar function, upper limb coordination, lower limb coordination, the peripheral nervous system, and upright stability, with a total score of 125 (FARA, 2023a). The mFARS is an updated version of the FARS, which better evaluates psychometric properties and reduces the number of tests, leading to a total rating obtained out of 93 (FARA, 2023a).

These different scales can be used in combination to identify abnormalities or severity of different symptoms which may present in FRDA, and have been used in the clinical trials which tested the compounds discussed in this review paper.

## 3 Molecular pathology of FRDA

It has been reported that the abnormal GAA repeat expansion is associated with the formation of non-B-DNA structures, R-loops, and sticky DNA (Wells, 2008; Hahn et al., 2010). Li *et al* reported that the process of transcription in FRDA patients is terminated prematurely at the upstream of the expanded GAA repeats and this also contributes to the formation of a novel, truncated and stable RNA (Li et al., 2022). They further observed that the early terminated transcript (*FXN-ett*) is subjected to alternative, non-productive splicing and does not participate in the synthesis of a functional frataxin (Li et al., 2022). The formation of these structures at the *FXN* locus may lead to the inhibition of RNA polymerase activity, thereby significantly slowing the rate of transcription (Hahn et al., 2010). It is therefore proposed that in such circumstance, cellular checkpoint processes may instigate the formation of heterochromatin (Hahn et al., 2010). Another thought-provoking mechanism proposed to expound the role of abnormal GAA repeat expansion in the formation of heterochromatin is the antisense transcription along the *FXN* locus. The formation of antisense transcript has been demonstrated in *Schizosaccharomyces pombe* where the antisense transcription generated a double stranded RNA (Grewal and Elgin, 2007). The RNAi machinery processes the dsRNA, and this may lead to the formation of heterochromatin along the locus of the gene (Grewal and Elgin, 2007). The presence of antisense transcription has been identified in other trinucleotide repeat expansion diseases such as myotonic dystrophy 1 (DM1) (Cho et al., 2005; Ladd et al., 2007). The generation of *FXN* antisense transcript 1 (FAST-1) has been associated with gene silencing mechanisms in FRDA patients, as evidence suggests that FAST-1 is not detected in unaffected individuals (Hahn et al., 2010). Furthermore, the low expression of CTCF (the proposed regulator of FAST-1) in FRDA patients, and the corresponding accumulation of FAST-1 in FRDA patients, underscores the role of antisense transcription in the formation of heterochromatin (Cho et al., 2005; De Biase et al., 2009; Hahn et al., 2010). The retention of intron 1 in the final *FXN* transcript has been suggested to be occasioned by the abnormal GAA repeat sequence. The GAA repeat sequence has been reported to bind to splicing factors such as those in the serine/arginine (SR)-rich protein family, and the proteins heterogeneous nuclear ribonucleoprotein (hnRNP) A1 and hnRNP A2, thereby rendering them incapable of splicing the introns during RNA processing (Baralle et al., 2008; Kumari and Usdin, 2012). This leads to the retention of mutated intron 1 in the final *FXN* transcript, hence affecting the expression of the frataxin protein. This was observed after a frataxin minigene (made up of *FXN* exon1, CMV promoter, part of intron 1 and the entire exon 2) transfected into COS7 cells and HeLa cells showed splicing deficiency when the GAA dominant strand was transcribed (Baralle et al., 2008).

## 4 Oxidative stress in FRDA

Oxidative stress has been implicated in the progression of neurodegenerative disorders such as Alzheimer’s Disease (AD), Parkinson’s Disease (PD), and Huntington’s Disease (HD), as well as FRDA (Andersen, 2004; Calabrese et al., 2005; Tamarit et al., 2016). The generation of oxidative stress in FRDA has been attributed to the low frataxin expression in FRDA patients and in disease models. The exact function of frataxin has not been completely elucidated, however observations from various experimental models where the downregulation or knock out of frataxin expression has been carried out may identify potential roles of the protein (Pandolfo, 2003; Busi and Gomez-Casati, 2012). The low expression of frataxin was observed to occasion mitochondrial labile iron accumulation, impair function of mitochondrial enzymes such as aconitase and succinate dehydrogenase, trigger downregulation of ARE-regulated genes, oxidative stress, and consequential cell death.

Several observations have pointed to a possible role of frataxin in iron-sulfur cluster formation. Frataxin interacts with the Nsf1/IscU complex (Tamarit et al., 2016). Nsf1 provides sulphur and IscU acts as a scaffold, which together promotes iron-sulfur cluster formation and enable them to act as cofactors for mitochondrial enzymes (Tamarit et al., 2016). Frataxin may stimulate the formation of these cofactors (Tamarit et al., 2016), and therefore reduced levels of frataxin may result in increased labile iron. Frataxin’s role in aconitase activity has also been investigated by several groups (Rötig et al., 1997; Bulteau et al., 2004; Tamarit et al., 2016; Mansilla et al., 2023). When cytosolic iron levels are low, cytosolic aconitase is converted into iron-responsive element binding protein (IRE-BP) (Rötig et al., 1997). IRE-BP binds to transferrin, protecting it from degradation, allowing it to transport more iron and increase iron levels (Rötig et al., 1997). This identifies one potential reason for reduced aconitase. However, frataxin’s role was further investigated in 2004, where it was shown to repair inactivated [3Fe-4S]^+^ cluster in aconitase (Bulteau et al., 2004). These findings were reported in various models and found to be an iron-dependent interaction (Mansilla et al., 2023), which further supports the idea that reduced frataxin leads to greater mitochondrial labile iron levels.

The excess accumulation of labile iron in the mitochondria of FRDA patients can react with the mitochondrial reactive oxygen species (ROS) that is inevitably present in these organelles due to occasional leakages of electron(s) from the electron transport chain (Brown et al., 2010; Zhao et al., 2019). These electrons reduce molecular oxygen to superoxide (O_2_^−^) anions (Zhao et al., 2019), the predominant ROS in the mitochondria. The mitochondrial superoxide dismutase 2 (SOD2) then converts the O_2_^−^ to hydrogen peroxide (H_2_O_2_). The H_2_O_2_ generated is further converted by catalase and/or glutathione peroxidase to water and oxygen (Zhao et al., 2019). In the event of iron accumulation in the mitochondria as established in FRDA (Sanchez-Casis et al., 1976; Lamarche et al., 1980), ferrous iron (Fe^2+^) reacts with H_2_O_2_ via the Fenton reaction to generate ferric iron (Fe^3+^) and hydroxyl radical (•OH) and hydroxyl (OH^−^): [Fe^2+^+ H_2_O_2_→ Fe^3+^+ •OH + OH^−^] (Koppenol and Hider, 2019; Rynkowska et al., 2020). The ferric iron formed also reacts with H_2_O_2_ to form hydroperoxyl (HOO•) and a proton (H^+^): [Fe^3+^+ H_2_O_2_→ Fe^2^ + HOO• + H^+^] (Koppenol and Hider, 2019; Rynkowska et al., 2020). The H_2_O_2_ is also capable of dissociating to form hydroperoxyl and hydroxyl radical: [2H_2_O_2_→ OH•+ HOO•+ H_2_O] (Koppenol and Hider, 2019). These radicals are highly reactive causing lipid, DNA and protein oxidation eventually leading to the death of neurons in the dorsal root ganglia and the dentate nucleus of FRDA patients (Angulo et al., 2021).

Polyunsaturated fatty acids (PUFAs) have been shown to be important for mitochondrial function, but are also very susceptible to oxidation, which can contribute to mitochondrial dysfunction (Tamarit et al., 2016). PUFAs, often referred to as the “good fats” are essential components of the cell involved in maintaining the structural integrity of the plasma membrane. PUFAs have also been reported to possess anti-inflammatory effects and are able to enhance mitochondrial function, reduce ROS generation and endoplasmic reticulum stress (Lepretti et al., 2018). Although PUFAs function to protect the cell from inflammatory and oxidative damage, the chemical composition of PUFAs (the carbon-hydrogen bonds) make them vulnerable to cleavage by reactive oxygen species (Nicolson, 2007). The cleavage of PUFAs leads to the generation of types of ROS which are very reactive with other PUFA molecules, thereby creating a vicious cycle with end products being ROS (Nicolson, 2007). The generation and accumulation of these ROS leads to the damage of the cellular membrane. Lipid peroxidation undergoes three steps: initiation, propagation, and termination (Gaschler and Stockwell, 2017). During initiation, free radicals are generated from lipids (R) [RH + •OH → •R + H_2_O], [•R + O_2_ → •ROO] (Gaschler and Stockwell, 2017). During propagation, radicals react with other substrates, forming more radicals [•ROO + RH → ROOH + •R], [ROOH → •ROO + H^+^] (Gaschler and Stockwell, 2017). Termination occurs when the radicals are quenched by antioxidants or by reacting with each other [•ROO + •ROO → RO-OR + O_2_] [•R + •R → R-R] (Gaschler and Stockwell, 2017). Altered lipid metabolism in FRDA has been reported in various models (Tamarit et al., 2016). In 2012, Martelli *et al.* found that reduced frataxin levels in liver conditional FRDA mouse model led to a buildup of lipid droplets and steatosis, which reduced the cristae and matrix material of the mitochondria (Tamarit et al., 2016). Schiavi *et al.* reported that in *Drosophila melanogaster,* frataxin deficiency results in increased fatty acid metabolism and higher rates of lipid peroxidation (Tamarit et al., 2016). Frataxin-deficient cardiomyocytes from rats also presented lipid droplets accompanied by decreased expression of PPARγ (peroxysome proliferators-activated receptor γ) (Obis et al., 2014). The activation of PPARγ is associated with glucose homeostasis, fatty acid oxidation, adipocyte differentiation in fat tissues and lipid storage (Rodríguez et al., 2020a). Whilst the mechanisms of altered lipid metabolism in FRDA are not completely understood it has been suggested that reduced PPARγ expression and its transcriptional regulator PGC-1α, could lead to reduced breakdown of lipids, specifically PUFAs, making them more readily available to undergo lipid peroxidation (Tamarit et al., 2016; Gaschler and Stockwell, 2017; Su et al., 2019).

Oxidative stress has been also associated with altered calcium (Ca^2+^) homeostasis in FRDA (Yang et al., 1999; Rodríguez et al., 2020b; Zhao et al., 2023). Ca^2+^ homeostasis is essential for optimal oxidative phosphorylation (Rodríguez et al., 2020b). The mitochondrial labile iron found in FRDA upregulates flux of Ca^2+^ into L-type Ca^2+^ channels, which promotes iron accumulation into cardiomyocytes, causing greater oxidative stress (Zhao et al., 2023). Treating FRDA fibroblast cell lines with the calcium chelator BAPTA-AM significantly rescued these cells from H_2_O_2_-induced oxidative stress (Yang et al., 1999). Furthermore, in *FXN*-silenced SH-SY5Y cell lines, Trolox, a lipid peroxidation scavenger and analogue of Vitamin E, improved Ca^2+^ uptake and reduced lipid peroxidation (Rodríguez et al., 2020b).

It is apparent that oxidative stress is one of the main drivers of mitochondrial dysfunction in FRDA. Furthermore, there are various different contributors and pathways leading to oxidative stress. It is therefore important to identify compounds which can effectively alleviate oxidative stress and its associated symptoms.

## 5 Antioxidants

**FIGURE 1:**
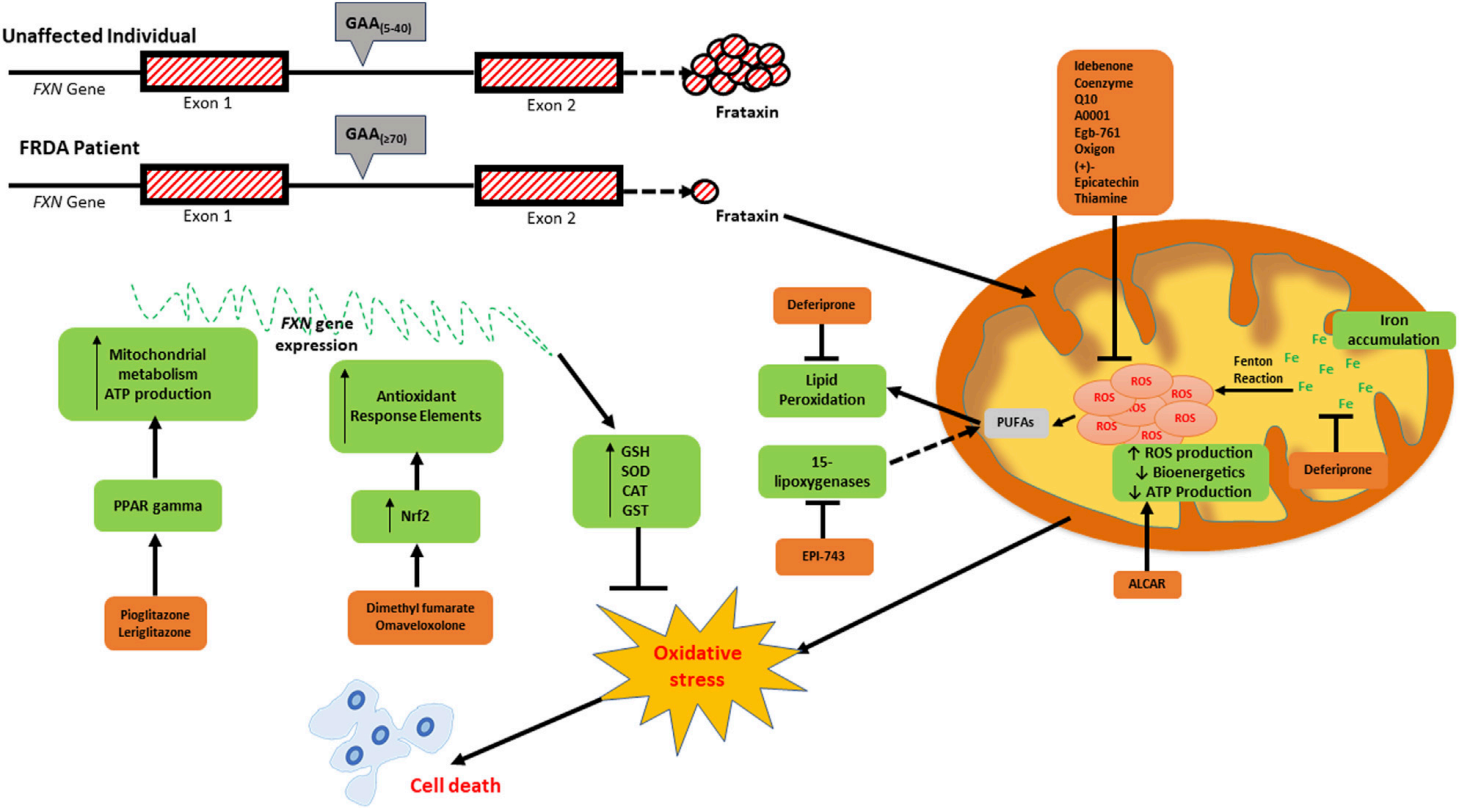
Schematic diagram depicting the consequences of frataxin deficiency and the various antioxidants which have been investigated as potential therapeutics for FRDA. The low expression of frataxin *inter alia* leads to mitochondrial iron accumulation due to reduced iron-sulfur cluster formation, increased generation of reactive oxygen species (ROS) via the Fenton reaction, reduced mitochondrial bioenergetics and ATP production, oxidative stress and consequential cell death. The elevated ROS levels can be targeted directly using ROS scavengers (idebenone, coenzyme Q10, A0001, EGb-761, oxigon, (+)-epicatechin, and thiamin). Deferiprone targets excess labile iron, whilst Acetyl-l-carnitine (ALCAR) increases mitochondrial bioenergetics and ATP production. EPI-743 and RT001 prevent ferroptosis by inhibiting both 15-lipogenases- and ROS-induced lipid peroxidation, respectively. Pioglitazone and leriglitazone function to activate PPAR gamma, which consequently leads to an increase in mitochondrial metabolism and ATP production. The upregulation of antioxidant response elements via the nuclear factor erythroid 2-related factor 2 (NRF2) pathway is activated by dimethyl fumarate (DMF) and omaveloxolone.

The therapeutic approaches currently being explored in FRDA are focused on either increase the levels of frataxin or mitigating the out-turn of frataxin loss. The role of antioxidants in FRDA therapy has become very important since oxidative stress has been recognised as one of the consequences of low frataxin levels. In targeting oxidative stress, several approaches are being explored to upregulate ARE-regulated genes and/or directly scavenge ROS. In this review, we will present the emerging antioxidant therapies in FRDA, those acting directly on ROS and those involved in the upregulation of antioxidant response elements and metabolic pathways in the mitochondria (Table 1). The specific processes of oxidative stress and mitochondrial biogenesis which these antioxidants work on are outlined in Figure 1.

**TABLE 1:** Summary of the antioxidants have been investigated as potential treatments for FRDA.

Summary of emerging antioxidant therapies for FRDA	
Upregulators of ARE-regulated enzymes and metabolic pathways in the mitochondria	
Dimethyl fumarate (DMF)	• Ester of fumaric acid, which is converted into monomethyl fumarate (MMF) upon administration
	• MMF activates NRF2 pathway and induces mitochondrial function
	• Already approved for the treatment of Multiple Sclerosis
	• Prodrug version of DMF (IXC-103) is set to undergo a phase I clinical trial with Ixchel Pharma
Omaveloxolone (RTA 408)	• Currently the only US FDA approved drug for the treatment of FRDA, under the tradename SKYCLARYS™
	• Activates AREs within antioxidant enzymes and enhances mitochondrial bioenergetics
	• The phase II MOXIe trial reported significant improvement in mFARS scores after 48 weeks
Resveratrol	• A plant polyphonic compounds identified in grapes, wines, and cocoa
	• It has been shown to reduce oxidative stress and increase phosphorylation of Akt and PI3K in the liver, and modulate downregulation of oxidative stress and inflammation markers (IL-1β, 1L-6, NF-ҡB) in muscle
	• Significant increases in frataxin expression after treatment have been shown in FRDA fibroblasts and YG8R mouse brain tissues, but not in FRDA iPSC-derived mesenchymal or neuronal cells
	• Currently undergoing a phase II clinical trial to assess tolerability
Pioglitazone	• PPARγ agonist, a receptor involved in glucose homeostasis and oxidation of fatty acids, activated by binding of PGC-1α, a transcription regulator for mitochondrial metabolism
	• Is currently used as a treatment for type II diabetes
	• Downregulation of PPARγ and PGC-1α after treatment has been shown *in vitro* and *in vivo* FRDA models, however results from phase III clinical trial estimated to conclude in 2013 are yet to be published
MIN-102 (Leriglitazone)	• A metabolite of pioglitazone which acts as a PPARγ agonist which exerted greater safety and tolerability over its predecessor in phase I
	• Results from phase II of the Minoryx Therapeutics trial found MIN-102 improved mitochondrial bioenergetics in FRDA
Acetyl-L-carnitine (ALCAR)	• Naturally produced in most mammalian tissues
	• Facilitate transport of long chain fatty acids into the mitochondria to be degraded for ATP synthesis
	• A phase I/II trial reported in 2005 found significant phosphocreatine recovery but no significance compared to placebo
	• Results from a clinical trial in FRDA patients estimated to finish in 2017 are yet to be published
Deferiprone	• An iron chelator capable of counteracting iron overload, crossing the blood-brain barrier and transferring iron to cellular iron acceptors
	• Currently used in treatment for thalassemia
	• A trial reported in 2011 found significant improvements in gait and echocardiographic results following treatment with deferiprone in FRDA patients in combination with idebenone
	• In 2014, a trial reported to find improvement in left ventricular mass at 20 and 40 mg/kg/d, however the greater dosage worsened FARS and ICARS
PTC-743 (Vatiquinone)	• A 15-lipoxygenase inhibitor, reducing ferroptosis and oxidative stress
	• The phase III MOVE-FA clinical trial by PTC Therapeutics found significant improvements in mFARS following 18 months of treatment
RT001	• A stabilised PUFA, protecting it from oxidative damage and reducing mitochondrial dysregulation
	• A phase II/III clinical trial by Retrotope did not meet its endpoints
ROS scavengers	
Idebenone (Catena)	• Molecule developed to improve electron transport within the mitochondria
	• A phase III clinical trial in the US found idebenone to be safe and tolerable and led to an improvement in neurological function
Coenzyme Q10 (Ubiquinone)	• Found in the inner mitochondrial membrane, facilitating electron transport between complexes
	• The results of a clinical trial published in 2008 found significant improvements in ICARS after 2 years of treatment in combination with vitamin E
Alpha-tocopherylquinone (A0001)	• A semi-synthetic compound similar in structure to idebenone and coenzyme Q10
	• Greater REDOX capabilities than coenzyme Q10, therefore exhibits greater ability to facilitate electron transport and reduce oxidative stress
EGb-761	• Obtained from Ginkgo Biloba
	• Has primary been investigated in AD and cognitive disorders
	• A phase II clinical trial by Ipsen in FRDA patients did not find any significant improvements
Indlole-3-propionic (Oxigon)	• Obtained as a by-product during the deamination of tryptophan
	• It has been reported that it can scavenge for hydroxyl radicals, and has shown neuroprotective effects *in vitro* and *in vivo* AD models
	• Has been shown to reduce oxidative stress resulting from amyloid-ß proteins and is approved for treatment of AD
	• A phase I clinical trial in FRDA patients did not find any significant improvements
(+)-Epicathechin	• Member of polyphenolic compounds identified in fruits, tea, cocoa, and red wine
	• (-)-Epicathechin is able to target AREs and improve mitochondrial biogenesis, however (+)-Epicathechin has a greater potency and half life than its negative enantiomer
	• A phase II (+)-Epicathechin clinical trial found improvements in left ventricular ejection fraction but not in FARS
Thiamine (Thamin, Vitamin B1)	• Cofactor for a number of enzymes involved in cellular bioenergetics
	• Has shown improvements in AD, PD, and HD
	• The results of a clinical trial reported in 2016 to find improved SARA scores after 80–930 days of treatment, as well as improved tendon reflexes and reduce interventricular septum thickness

### 5.1 Upregulation of antioxidant response elements and metabolic pathways in the mitochondria

#### 5.1.1 NRF2 inducers

NRF2 (Nuclear factor Erythroid 2-related Factor 2) is a transcription factor involved in the expression of antioxidant enzymes which contain the ARE (Li et al., 2019). Under ordinary conditions, NRF2 is bound to the inhibitor Kelch-like ECH-associated protein 1 (KEAP1) within the cytoplasm (Nguyen et al., 2003; Kavian et al., 2018; Pi et al., 2019; Rodríguez et al., 2020a). KEAP1 is therefore capable of constantly degrading NRF2 in the cytoplasm (Niture et al., 2014; Li et al., 2019). This NRF2 degrader operates as an adapter for CUL3/RBX1 E3 ubiquitin ligase-mediated degradation of NRF2. In the presence of ROS, NRF2 dissociates from KEAP1 and undergoes translocation into the nucleus, where it binds to the AREs, the enhancer sequences, of antioxidant enzymes (Nguyen et al., 2003; Pi et al., 2019; Rodríguez et al., 2020a). This activates genes coding for antioxidant enzymes to counter the effects of oxidative stress (Niture et al., 2014; Li et al., 2019). The AREs in these enzymes have the common promoter sequence 5′-TGACNNNGC-3′ (Pi et al., 2019; Rodríguez et al., 2020a). NRF2 has since been described as the principal regulator of antioxidant response in cells. Since oxidative stress has characterised most of the pathologic events in neurodegenerative diseases, activation of NRF2 pathway has been proposed as a major therapeutic strategy to obstruct disease onset, blunt disease progression and meliorate symptoms of these neurodegenerative diseases (Petrillo et al., 2019). Impaired expression of NRF2 has been reported in FRDA patients and various disease models (Paupe et al., 2009; Anzovino et al., 2017). Shan *et al.* have reported that low levels of frataxin leads to the reduced expression of NRF2 and antioxidants present in the dorsal root ganglia of FRDA mouse models (Shan et al., 2013). This has therefore driven research into drug candidates capable of activating NRF2 to ostensibly increase NRF2 expression in FRDA patients (Petrillo et al., 2019).

##### 5.1.1.1 Dimethyl fumarate (DMF)

Dimethyl fumarate (DMF) is a drug which has been approved for the treatment for multiple sclerosis and psoriasis (Sator et al., 2019; Lategan et al., 2021). DMF has been established to exert its therapeutic functions by activating the NRF2 pathway thereby inducing mitochondrial function; the exact biological process found to be deficient in FRDA. It was therefore hypothesised that DMF may be beneficial to FRDA patients. DMF is an ester of fumaric acid and following its administration, DMF is converted to monomethyl fumarate (MMF) which then upregulates the NRF2 pathway. The treatment of lymphoblasts obtained from FRDA patients was found to significantly increase frataxin gene and protein expression (Jasoliya et al., 2019) and also dose-dependently increase mitochondrial function in FRDA mouse models (Hui et al., 2020). A prodrug version of DMF which has been developed by Ixchel Pharma is set to be put up for phase 1 clinical trials (FARA, 2023b).

##### 5.1.1.2 Omaveloxolone (RTA 408)

Omaveloxolone (RTA 408, Skyclarys™) or Omav is a semisynthetic oleanane triterpenoid developed by Reeta Pharmaceuticals to target the upregulation of the NRF2 pathway (FARA, 2023b). Omav activates NRF2 and truncates the inhibitory effects of KEAP1 over NRF2 (Robledinos-Antón et al., 2019). The antioxidant activities of Omav have been reported to be via the activation of ARE-regulated genes as well as enhancement in mitochondrial bioenergetics (Rodríguez et al., 2020a). These effects have made Omav a potential candidate for FRDA therapy, since the downregulation and reduced expression of NRF2, coupled with defective mitochondrial bioenergetics have been widely reported in FRDA patients. A study conducted by Abeti et al. (2018) reported that Omav was able to protect against mitochondrial depolarisation, cell death and promoted mitochondrial respiration in YG8R and KIKO mouse models, and also fibroblast cells from FRDA patients. A significant improvement in Complex I activity in these *in vitro* and *in vivo* models was also reported in this study (Abeti et al., 2018). A two-part multicenter, double-blind, randomised, placebo-controlled study designated the MOXIe (NCT02255435, Reata Pharmaceuticals Inc.) was conducted to evaluate the safety and efficacy of Omav in FRDA patients (Ghanekar et al., 2019). The outcome of the first part of the trial revealed that Omav was well tolerated with mild adverse events. A dose-dependent improvement in the mFARS score of the patients was also recorded. The phase II of the MOXIe trial to assess the safety and efficacy of 150 mg of Omav in genetically diagnosed FRDA patients was also reported to have met its primary endpoint which is a significant change from baseline in mFARS score at 48 weeks (ClinicalTrials, 2021b). The trial is currently due to meet its secondary endpoint in December 2024 (ClinicalTrials, 2021b). The US Food and Drug Administration (FDA) has approved Omav as the first and only FDA approved treatment for FRDA patients (FARA, 2023b). Specifically, the treatment has been approved for those over the age of 16 and not suffering from severe hepatic impairment, as it may cause reversible increases in aspartate aminotransferase (AST) or alanine aminotransferase (ALT) (Lee, 2023).

#### 5.1.2 Resveratrol

Resveratrol is a plant polyphenolic compound found in grapes’ skin and seeds, red and white wine and cocoa (Tian and Liu, 2020). This compound has been reported to have antioxidant, anti-inflammatory, antiapoptotic and neuroprotective effects (Grinan-Ferre et al., 2021; Huo et al., 2021). Resveratrol has been reported to have a wide range of effects in different organs of the body. In the liver, it reduces oxidative stress and cytokine-mediated inflammation, increases mitochondrial biogenesis, reduces AMPK mediated fatty acid synthesis and downregulates gluconeogenesis whilst upregulating glycogen synthase activity and phosphorylation of Akt and P13K (Pannu and Bhatnagar, 2019). In the muscles, resveratrol has been reported to be involved in the upregulation of GLUT-4 mediated glucose transport, mitochondrial biogenesis, SIRT-1 and AMPK activation, and downregulation of oxidative stress and inflammation biomarkers (IL-1β, 1L-6, NF-ҡB) (Pannu and Bhatnagar, 2019). The effects of resveratrol in the pancreas have been reported to lead to increase in ß-cell number and survival, prevent apoptosis by downregulating caspase and PARP cleavage, and prevent ß-cell damage by exerting antioxidant defences (Pannu and Bhatnagar, 2019). In the adipose tissues, resveratrol has been found to downregulate AMPK-α phosphorylation and SIRT-1 expression, reduce microphage infiltration and lipogenic enzymes levels (Pannu and Bhatnagar, 2019). The effects of resveratrol were corroborated using *in vitro* and *in vivo* models where it was reported that resveratrol treatment of Hela cells, lymphoblasts, fibroblasts from FRDA patients, and brain tissues from YG8R FRDA mouse models lead to a significant expression of frataxin (Li et al., 2013; Georges et al., 2019). It was however reported that resveratrol had a little or no effects when treated with mesenchymal and neuronal cells differentiated from patient-derived induced pluripotent stem cells (iPSCs) (Georges et al., 2019). These results somewhat generated some uncertainties with respect to the true effects of resveratrol in FRDA patients (Georges et al., 2019). An open-label study sponsored by FARA was conducted to determine the safety, tolerability, and efficacy of two doses (1 g and 5 g) of resveratrol in FRDA patients (FARA, 2023b). It was reported that resveratrol had no effects on the frataxin expression in the participants (Yiu et al., 2015). However, there was an improvement in neurological symptoms (thus improved FARS and ICARS scores) in participants who received 5 g of the drug (Yiu et al., 2015). Major adverse events such as diarrhoea and abdominal pain were reported in the high dose group (Yiu et al., 2015). The adverse events recorded have called for modifications in resveratrol to improve its tolerability. A phase II study sponsored by Jupiter Orphan Therapeutics, Jupiter, FL and Murdoch Children’s Research Institute (MCRI), Melbourne, Australia, is currently underway to assess the tolerability and efficacy of micronised resveratrol, and is estimated to be completed in February 2024 (ClinicalTrials, 2021a; FARA, 2023b).

#### 5.1.3 Pioglitazone

Pioglitazone is a drug which is currently being used for the treatment of type II diabetes. Apart from its therapeutic role in diabetic patients, pioglitazone has been reported to upregulate the expression of enzymes (such as superoxide dismutases) involved in metabolic pathways in the mitochondria via the activation of PPARγ (Marmolino et al., 2010). The activation of PPARγ depends on the binding of PGC-1α; a transcriptional regulator mainly involved in mitochondrial metabolism. Pioglitazone is an agonist of PPARγ hence the binding of pioglitazone to its ligand leads to the upregulation of antioxidant responses, a phenomenon which has been reported to be deficient in FRDA patients. Several studies have shown the downregulation of PPARγ and PGC-1α in various cell types obtained from FRDA patients and animal models (Coppola et al., 2009; Marmolino and Acquaviva, 2009), a situation suggesting the likely importance of pioglitazone in FRDA therapy. A randomised, double-blind controlled trial was conducted to evaluate the effects of pioglitazone on neurologic function (ClinicalTrials, 2013). The study was completed in 2013 but, as of January 2024, the outcome has still to be published (ClinicalTrials, 2013), presumably due to lack of efficacy of pioglitazone in FRDA patients.

#### 5.1.4 MIN-102 (leriglitazone)

Leriglitazone is a metabolite of pioglitazone and a PPARγ agonist which has been reported to have a good bioavailability in the central nervous system coupled with a better safety profile. Interestingly, the outcome of a phase one clinical trial indicated that MIN-102 was well-tolerated and its therapeutic effect was way above what had been reported in preclinical studies (Poli et al., 2020). A phase two randomised, double-blind, placebo-controlled study was initiated by Minoryx Therapeutics to assess the effects of MIN-102 on biochemical, neurophysiological, imaging and clinical biomarkers in FRDA patients. It was reported from this study that MIN-102 had a significant impact on relevant disease biomarkers in the central nervous system of all participants. MIN-102 was able to interact with PPARγ receptors as observed from the assessment of adiponectin in FRDA patients after the trial. The study also reported that MIN-102 modulated the defective frataxin pathway in the patients thereby restoring the bioenergetic defects in FRDA (Meya et al., 2020; Poli et al., 2020). A confirmatory study is being planned to further assess the therapeutic effects of MIN-102 in FRDA patients and other neurodegenerative diseases.

#### 5.1.5 Acetyl- L-Carnitine (ALCAR)

L-carnitine (LC, β-hydroxy-y-trimethylaminobutyric acid) is a naturally occurring compound present in most mammalian tissues, and has been reported to be involved in the transport of long chain fatty acids into the mitochondria where they are degraded by β-oxidation to generate ATP (Ribas et al., 2014). Research shows that L-carnitine and its acetylated analogue, acetyl-l-carnitine (ALCAR) are present in human tissues including the brain as well as in peripheral blood (Ferreira and McKenna, 2017). Over the past few years, there has been a growing interest in the therapeutic potentials of L-carnitine and ALCAR in conditions associated with nervous system injury and degeneration (Zanelli et al., 2005; Alves et al., 2009; Zhang et al., 2012). A placebo-controlled triple-phase crossover study was conducted in FRDA patients for 4 months to evaluate mitochondrial ATP production and neurologic improvements (Schöls et al., 2005). Phosphocreatine recovery was used as the measure of mitochondrial ATP production. Participants were given 3 g/day L-carnitine and 6.75 g/day creatine and it was reported that there was a significant improvement in mitochondrial ATP production amongst group who received L-carnitine (Schöls et al., 2005). However, creatine had no significant effect as there was no change in ICARS score and echocardiographic variables. The outcome of this trial therefore suggests the potential of L-carnitine as a therapy for FRDA (Schöls et al., 2005). An open-label pilot study of the acetylated analogue of ALCAR was sponsored by University of Florida with the aim to evaluate the impact of ALCAR on the heart and other ataxia gait symptoms of FRDA patients (ClinicalTrials, 2016a). The participants were given 2 g/day ALCAR for 24 months and the study was estimated to be completed in 2017 (ClinicalTrials, 2016a). The outcome of this study has not yet been published.

#### 5.1.6 Deferiprone

Deferiprone (Ferriprox^®^) is an iron chelator which has been approved in Europe and the United States for the treatment of thalassemia (Morales et al., 2022). FRDA leads to the accumulation of labile iron within the mitochondria (Alsina et al., 2018). This has led to the application of iron chelators as a potential therapeutic target. Deferiprone is unique among iron chelators as it can successfully counteract iron overload, cross the blood brain barrier and other lipophilic membranes, as well as transferring iron to transferrin, including other cellular iron acceptors (Sohn et al., 2008; Botzenhardt et al., 2017). Preclinical study conducted by Kakhlon et al. (2008) revealed that the treatment of frataxin-deficient induced HEK-293 cells with clinical concentrations of deferiprone successfully chelated the mitochondrial labile iron identified to propagate oxidative stress. These translated into high production of ATP and oxygen consumption, reduced damage to mitochondrial DNA and reactivation of impaired mitochondria membrane and its associated functions (Kakhlon et al., 2008). A randomized, double-blind, placebo-controlled study was conducted to investigate the safety, tolerability, and efficacy of deferiprone in FRDA patients (Pandolfo et al., 2014). Participants received 20, 40 or 60 mg/kg/d or placebo and it was reported that the 60 mg/kg/d treatment was discontinued due to it exacerbating the ataxia symptoms in 2 patients (Pandolfo et al., 2014). The 20 and 40 mg/kg/d treatments were found to be tolerable in patients coupled with a significant reduction in left ventricular mass index as compared to an increasing left ventricular mass index in the placebo arm. However, neither the 20 mg/kg/d nor 40 mg/kg/d had a positive impact on FARS or ICARS score. It is worth noting that whilst the 20 mg/kg/d had no impact on FARS score when compared to the placebo arm, the 40 mg/kg/d worsened the FARS and ICARS score of participants (Pandolfo et al., 2014). The inconsistent outcomes may be due to non-specific iron chelation therapy which was worsened when higher doses were used. In fact, a key parameter omitted in the trials with deferiprone was that the iron chelator was not targeted to the mitochondria, so effectively only a small fraction of the compound delivered would accumulate in the principal site of damage under these conditions. This often forces clinical trials to use higher doses of drug that lead to unintended side-effects and cytotoxicity. Targeting active molecules to the mitochondria is likely to improve their effectiveness against oxidative insult and hence be beneficial for FRDA patients due to reduced off-target therapy, increased specificity and lower therapeutic dosage required of the targeted chelator (Reelfs, et al., 2019; Cilibrizzi, et al., 2023).

#### 5.1.7 PTC-743

PTC-743/Vatiquinone (previously known as EPI-743) is a compound found to impair the activities of 15-lipoxygenase; a principal enzyme regulator of inflammation, ferroptosis and oxidative stress (Kahn-Kirby et al., 2019). The antioxidant activities of PTC-743 have been described to be from a 1000 to 10000-fold higher than resveratrol, idebenone or coenzyme Q10 (Shrader et al., 2011). In 2013, a double-blind randomized controlled study was sponsored by Edison Pharmaceuticals to evaluate the safety and efficacy of EPI-743 in FRDA patients (Zesiewicz et al., 2018b). It was revealed from the trial that EPI-743 was safe and well-tolerated amongst participants. The assessment of the primary endpoint (a measure of vision) at 6 months of study revealed no significant impact between the EPI-743 treated arm and placebo arm (Zesiewicz et al., 2018b). However, assessment using FARS revealed a trend in neurological improvement. Results from the entire duration of study (24 months) revealed a significant improvement in FARS score indicating a significant impact on neurologic function of the EPI-743 when compared to the place group (Zesiewicz et al., 2018b). In 2021, PTC Therapeutics initiated a phase 3 study (MOVE-FA) study which sought to evaluate the effects of PTC-743 in children and young adults with FRDA. The duration of this study is 18 months with primary endpoints being a significant improvement from baseline in mFARS and significant improvement in ambulation and daily activities being the secondary endpoints (FARA, 2023b). PTC-743 has been granted an orphan drug status by the United States Food and Drug Administration for FRDA (FARA, 2023b).

#### 5.1.8 RT001 (deuterated fatty acids)

PUFAs are susceptible to high rates of oxidation, leading to greater mitochondrial dysfunction (Tamarit et al., 2016). The development of RT001 sought to stabilise the PUFAs, thereby protecting the plasma membrane integrity of the cell. The strategy being adopted in this regard is to develop mimetics of PUFA by replacing the hydrogen (^1^H) with the stable isotope deuterium (^2^H) (Cotticelli et al., 2013). This approach has been reported to stabilise the PUFA hence making it resistant to lipid peroxidation (Cotticelli et al., 2013). A preclinical study conducted by Abeti et al. (2015) revealed that deuterated PUFAs (dPUFAs) significantly prevented cell death, lipid peroxidation in FRDA cells *in vitro*. A phase I/II double-blind comparator-controlled study was conducted by Retrotope to assess the safety and efficacy of RT001 in FRDA patients revealed that the drug was safe and well-tolerated in participants coupled with cardiopulmonary improvements (Zesiewicz et al., 2018a). Retrotope conducted a phase III clinical trial to further establish the long-term safety and efficacy of RT001, which was reported not to meet the endpoints of the trial (FARA, 2023b).

### 5.2 ROS scavengers

ROS represent a set of free radicals and molecules carrying an unpaired valence electron obtained from oxygen (Sies et al., 2022; Zhao, et al., 2022). Under normal physiological condition, there is a fine balance between ROS production and ROS elimination. However, an imbalance is created following the increase in ROS production and significant decline in ROS elimination. This leads to accumulation of ROS and subsequent pathologies as reported in neurodegenerative diseases such as FRDA, PD, AD, Amyotrophic Lateral Sclerosis (ALS) (Bhatt et al., 2021; Yeung et al., 2021). The role of ROS has also been reported in cardiovascular diseases (Wang et al., 2021; Zhou et al., 2021). The generation of ROS can be from exogenous and or endogenous sources. The exogenous sources of ROS include radiation, xenobiotics and pollutants. The generation of ROS endogenously stems from the metabolic processes taking place within the cell; oxidation phosphorylation (from the leakage of electrons in the electron transport chain) (Chen et al., 2003; Murphy, 2009), oxidation catalysed by metals and reaction of NADPH oxidase as reported in the respiratory burst of phagocytes (Robinson, 2008). ROS scavengers act to neutralize and prevent the accumulation of ROS in cells.

#### 5.2.1 Idebenone (Catena)

Idebenone (Catena^®^) is a small molecule developed to enhance the electron balance by facilitating electron transport in the mitochondria. Whilst this small molecule has received a conditional approval as a treatment modality for FRDA in Canada (Santhera Pharmaceuticals, Liestal, Switzerland) (Hawi et al., 2012), other countries including the United States areyet to consider the approval of Catena as a treatment for FRDA. Phase IIIb Double-Blind, Randomised, Placebo-Controlled Study of PROTI (Patients Reported Outcomes in Friedreich’s Ataxia Patients After Withdrawal from Treatment with Idebenone) was to see if the patients can identify the treatment they received during the trial. The study involved 29 participants. Participants weighing 45 kg or less received 1350 mg/day of Idebenone with meals, whilst those weighing 45 kg or more received 2,250 mg/day Idebenone with meals. For the placebo patients, those weighing 45 kg or less received 3 tablets 3 times per day with meals, whilst those weighing 45 kg or more received 5 tablets 3 times per day with meals. The exact outcome of this study is still pending. A phase III clinical trial of Catena^®^ in the United States was reported not to have a statistically significant benefit amongst participants, although the study did indicate that Catena^®^ was safe and well-tolerated in study participants (Lynch et al., 2010). Improvement in various rating scales were also reported from this study as patients who received Catena^®^ showed 2.5 points improvements on their mean ICARS, 1.6 points on FARS as compared to the placebo group reported to have 1.3 points, 0.6 points on ICARS and FARS respectively (Lynch et al., 2010). The effects of Idebenone on cardiac functions in FRDA patients were investigated in a phase III (IONIA) randomized, double-blind, controlled trial involving 70 paediatric patients. Patients received either (450/900 mg/day or 1350/2250 mg/day Idebenone) or placebo for 6 months. It was reported from the study that Idebenone was unable to reduce left ventricular hypertrophy and there was no improvement in the cardiac functions of the patients at the end of the study (Lagedrost et al., 2011). A phase III IONIA open-label extension study was conducted to investigate the effects of Idebenone on neurological function in 68 pediatric patients. It was reported from the study that the 1350/2250 mg/day Idebenone for 12 months lead to an improvement in the neurological function of the pediatric patients (Lynch et al., 2010). The outcomes of Idebenone in patients have depicted some improvements however, these improvements have not been consistent and statistically significant. This therefore makes the approval of Idebenone (Catena^®^) in the United States sceptical. Idebenone is however available for purchase as a supplement/nutraceutical in the United States.

#### 5.2.2 Coenzyme Q10 (Ubiquinone)

Ubiquinone is a small hydrophobic molecule present in the inner mitochondrial membrane. Ubiquinone is involved in the carrier of electrons from complexes I and II to complex III of the electron transport chain during oxidation phosphorylation (Orsucci et al., 2011; Zhao et al., 2019). Due to its important role in the respiratory chain and mitochondrial bioenergetics, it has been widely used in the treatment of mitochondrial disorders (Orsucci et al., 2011). A preclinical study conducted by Jauslin et al. (2003) reported that Coenzyme Q10 and its analogues have been able to prevent cell death in fibroblasts obtained from FRDA patients. The therapeutic effects of Coenzyme Q10 was evaluated in 10 FRDA patients in an open-label study. Participants were given a combination of Coenzyme Q10 (400 mg/day) and Vitamin E (2,100 IU/day) (Lodi et al., 2001). The evaluation of neurological and echocardiographic outcomes indicates no consistent benefits after 6 months of treatment. However, improvements in cardiac and skeletal muscle bioenergetics were observed hence authors suggest the lack of neurological and echocardiographic improvements to be due to the short duration of study before evaluation (Hart et al., 2005). They then continued to monitor the patients to ascertain if the improvements will be sustained for 47 months. After this duration of treatment, a significant improvement in skeletal and cardiac muscle bioenergetics were reported. The ICARS scores recorded in the earlier assessment were no different from what was recorded following 47 months of treatment notwithstanding the consistent decline in gait and posture symptoms (Lodi et al., 2001; Hart et al., 2005). Schapira *et al* conducted a randomized double-blind trial involving 50 FRDA patients who were given a combination of high dose CoQ10/Vitamin E for 2 years (Cooper et al., 2008). It was reported after the study that almost half (49%) of participants had a slowed degeneration coupled with an increased clinical improvement, thereby having positive impact on their ICARS scores (Cooper et al., 2008). This outcome suggests a possible breakthrough in FRDA therapy using combinatorial therapy of antioxidants.

#### 5.2.3 Alpha-tocopherylquinone (A0001)

Alpha-tocopherylquinone:2-[(3R,7R,11R)-3-hydroxy-3,7,11,15-tetramethyl-hexadecyl]-3,5,6 trimethylcyclohexa-2,5-diene-1,4-dione) is a semi-synthetic compound with structural similarity to Coenzyme Q10 and Idebenone. A0001 has been reported to possess more redox capabilities as compared to CoQ10 hence expected to be more efficacious in mitigating mitochondrial oxidative stress, lipid peroxidation and to enhance the transfer of electrons in a dysfunctional respiratory chain (Hawi et al., 2012; Rodríguez et al., 2020a). A0001 is therefore being developed as a treatment modality for Friedreich’s ataxia patients and mitochondrial encephalomyopathy, lactic acidosis, and stroke-like episodes (MELAS) where mitochondrial redox pathologies have been identified (Hawi et al., 2012). Hawi et al. (2012) conducted a single ascending-dose study to evaluate the pharmacokinetic profile of A0001 in 10 participants. They reported that A0001 was well-tolerated with no major adverse effects amongst participants, and further suggested A0001 to be taken with food as this enhances the pharmacokinetics of A0001. Based on the results obtained from *in vitro study* by Lynch et al. (2012) where A0001 rescued I154F frataxin point-mutation model from cell death, a double-blind placebo-controlled trial involving 45 patients was initiated by them (Calmels et al., 2009). It was reported from the double-blind placebo-controlled trial that although A0001 was well-tolerated amongst participants with no major adverse reactions, there was no significance difference between the A0001 and placebo groups with respect to glucose tolerance (Lynch et al., 2012). Interestingly, a significant improvement in neurologic functions was recorded as the placebo group improved their FARS score by 2.0 as compared to participants on A0001 who had a 4.9 score on FARS (Lynch et al., 2012). No further studies have been conducted to assess the reproducibility or consistency of this outcome. However, the US Food and Drug Administration granted an orphan drug status to A0001 as a treatment modality for inherited mitochondrial disorders (Hawi et al., 2012).

#### 5.2.4 EGb-761

EGb-761 is a Ginkgo Biloba extract and it is the most investigated standardised herbal preparations for the management of disorders associated with cognition and AD (Kandiah et al., 2021; Mansour et al., 2021). The faith in Ginkgo Biloba extract stemmed from the observed antioxidant and neuroprotective effects in several *in vitro* (Zhou and Zhu, 2000; Guidetti et al., 2001) and *in vivo* studies (Zhang, et al., 2000; Ferrante et al., 2001). The positive outlook of Ginkgo Biloba extract in patients with AD, and the similarities in redox pathologies between FRDA and AD lead to a phase II, randomised, placebo-controlled double-blind trial conducted by Ipsen in France involving 22 FRDA patients. This trial was to assess the efficacy of EGb-761 in FRDA patients. Participants in the EGb-761 arm received 120 mg twice a day for 12–14 weeks. After the study, there was no significant clinical difference between the EGb-761 arm and placebo arm. Authors stated the number of participants was not enough to carry out any statistical analysis whatsoever hence the study was considered null and void (Rodríguez et al., 2020a).

#### 5.2.5 Indole-3-propionic acid (oxigon)

Indole-3-propionic acid (also known as SHP622, VP-20629, OXIGON or OX1) is a by-product from the deamination of the amino acid tryptophan which has been reported to have antioxidant, anti-inflammatory and neuroprotective properties (Garcez et al., 2020; Owumi et al., 2022). It is reported to be found in the cerebrospinal fluid and human plasma and has ability to scavenge for hydroxyl radicals thereby attenuating lipid peroxidation and exerting its neuroprotective effects (Owumi et al., 2022). The neuroprotective effects of indole-3-propionic acid has been reported in several *in vitro* and *in vivo* models of AD (Rodríguez et al., 2020a; Owumi et al., 2022). This drug has been approved for the treatment of AD based on its therapeutic effects in mitigating oxidative stress occasioned by beta-amyloid proteins (Bendheim et al., 2002). Based on the similarities in redox pathology between AD and FRDA, 46 FRDA patients were recruited for a phase I study to evaluate the safety and pharmacokinetic profile of Indole-3-propionic acid. Following trial completion, it was reported that the drug was well-tolerated with no major adverse effects however, no major benefit was observed after or during of the study (ClinicalTrials, 2016b; Rodríguez et al., 2020a).

#### 5.2.6 (+)-epicatechin

Epicatechin belongs to a group of polyphenolic compounds found in fruits, tea, cocoa and red wine, and has been reported to possess antioxidant properties (Fraga et al., 2018; Qureshi et al., 2021). The negative enantiomer of epicatechin; (-)-epicatechin has been observed to regulate cellular ARE containing enzymes via the NRF2 pathway and facilitate mitochondrial biogenesis via SIRT1/NRF1 pathway (Moreno-Ulloa et al., 2015; Fraga et al., 2018). This natural flavonoid has been reported to decrease the generation of ROS, enhance cardiac function and skeletal muscle regeneration (Yamazaki et al., 2010), and stimulates neuronal regeneration (Shay et al., 2015). The positive enantiomer of epicatechin; (+)-Epicatechin has been reported to have a higher potency than (-)-epicatechin with respect to mitochondrial biogenesis and cardiac function due to the higher half-life of the positive enantiomer as compared to the negative enantiomer of epicatechin. (Ramirez-Sanchez et al., 2014; Moreno-Ulloa et al., 2018). Based on the positive outlook of (+)-epicatechin, a phase I study was rolled out to evaluate the pharmacokinetic and pharmacodynamic profile of this flavonoid. It was reported from the study that (+)-epicatechin was well-tolerated with no major adverse events amongst healthy and diabetic participants (Moreno-Ulloa et al., 2018). A phase II open-label study was conducted amongst 10 FRDA patients to evaluate the safety and efficacy of (+)-epicatechin. After the duration of the study, it was again reported that the compound was well-tolerated with no major adverse events coupled with an improvement in the left ventricular ejection fraction. However, no improvement of FARS was recorded after the 24 weeks study was complete (Qureshi et al., 2021).

#### 5.2.7 Thiamine (Thamin, vitamin B1)

Thiamin is a cofactor of number of enzymes involved in cell bioenergetics, and a pharmacological agent which has been reported to improve cognitive functions in patients with neurodegenerative disease such as AD, HD, and PD (Blass et al., 1988; Meador et al., 1993; Nguyê, 2012; Costantini et al., 2013; Lương and Nguyễn, 2013). The reduced levels of thiamin and its coenzyme form, thiamin diphosphate (ThDP), in the aged have been reported. In AD patients, low levels of ThDP were observed in post-mortem cortex samples as compared to age-matched controls (Bettendorff et al., 1997; Gangolf et al., 2010). The role of thiamin as a radical scavenger was reported as thiamin deficiency has been found to result in defects in oxidative metabolism, neuroinflammation, increased levels of oxidative stress and selective loss of neurons in the nervous tissues (Liu et al., 2017). These pathologic characteristics of thiamin deficiency have widely been reported in FRDA patients, and the low levels of thiamin in the cerebrospinal fluid of FRDA further underscores the pathologic similarities associated with thiamin deficiency in FRDA, AD, HD and PD (Pedraza and Botez, 1992; Botez and Young, 2001). In view of these pathologic similarities, an open-label study was conducted to evaluate the impact of thiamin on neurological symptoms, echocardiographic variables and plasma levels of *FXN* gene expression. Thirty-four FRDA patients were given 100 mg intramuscular thiamin twice a week for 80–930 days (Costantini et al., 2016). Assessment was done intermittently until the end of the study, and it was reported that thiamin significantly improved SARA score, alleviated swallowing difficulties and recovery of tendon reflexes and thickness of the interventricular septum (Costantini et al., 2016). Thiamin was not further considered as a potential therapy for FRDA because there was no placebo group during the open-label study and no further studies has been done on its therapeutic potentials (Costantini et al., 2016; Rodríguez et al., 2020a).

### 5.3 Elamipretide

Elamipretide (ELAM, SS-31, Bendavia, ELViS-FA, MTP-131) is a mitochondria-targeted peptide, capable of localising to the inner mitochondrial membrane (IMM) where it associates with cardiolipin and modulates mitochondria function (ClinicalTrials, 2022; Karaa et al., 2023; Pharaoh et al., 2023). This leads to increased production of supercomplexes, greater membrane stability, reducing ROS levels and elevating ATP synthesis (Karaa et al., 2023). Elamipretide treatment was also shown to increase ADP sensitivity *in vivo* and decrease ROS production *in vitro* (Pharaoh et al., 2023). This has led to clinical trials investigating the potential therapeutic effect of elamipretide.

A phase III clinical trial (MMPOWER-3) investigating elamipretide in primary mitochondrial myopathy (PMM) was finished in 2020 (ClinicalTrials, 2017; Karaa et al., 2023). It was reported that, whilst the drug was well tolerated by participants, there were no significant differences in walking (evaluated using the 6-min walk test) or fatigue (evaluated using the PMM Symptom Assessment Total Fatigue Score) (ClinicalTrials, 2017; Karaa et al., 2023). In 2022, a phase I/II clinical trial was set up to investigate elamipretide as a potential therapeutic target for FRDA (ClinicalTrials, 2022). The primary outcome is expected to be completed in July 2024, and aims to investigate if there are improvements in high contrast visual acuity (ClinicalTrials, 2022). The secondary outcome will aim to investigate cardiac strain, fibrosis, and stroke volume (ClinicalTrials, 2022).

## 6 Discussion and conclusions

This review sought to discuss antioxidants being considered as therapeutic modality for FRDA ostensibly to blunt disease progression and improve the symptoms of FRDA. Due to the pathologic implication of oxidative stress in FRDA, attempts have been and are being made to develop drugs which can counteract the impact of oxidative stress in FRDA. Preclinical studies of most of these drugs were quite satisfying, however, their impact in patients as reported from clinical trials have not been really encouraging. Notwithstanding the non-performance of some of these antioxidants in clinical trials, it is worth noting that a couple of them have also shown some remarkable results in patients during clinical trials). The non-performance of most of the drugs in clinical trials could be attributed to a host of factors. FRDA is a rare disease, hence the challenge of recruiting large number of patients to impact clinical significance is one which cannot be underestimated. The impact of age of disease onset, length of GAA repeats and stage of disease progression on the outcome of clinical trials have not been clearly demonstrated. The interplay between genetics and epigenetics in oxidative stress have also not been clearly established in the case of FRDA. In addition to this, there are various factors contributing to oxidative stress in FRDA, including mitochondrial labile iron, NRF2 expression, lipid peroxidation, and Ca^2+^ homeostasis, which means therapeutics may need to combat a number of these factors to overcome oxidative stress. Hence, the failure of some of these antioxidants in clinical trials may not be necessarily due to their pharmacological effects.

It is worth noting that none of the antioxidants reviewed had a significant effect on frataxin gene and protein expression in FRDA patients during their respective clinical trials. However, a vast majority of the antioxidants had a significant improvement in neurological and cardiac functions as measured by FARS, SARA or mFARS score and left ventricular ejection fraction respectively (Ramirez-Sanchez et al., 2014; Yiu et al., 2015; Lynch et al., 2021; Qureshi et al., 2021). Based on the underlying cause of FRDA and its subsequent transition into reduced frataxin gene and protein expression, drugs which may not affect frataxin gene and protein expression may not be able to completely provide the needed therapeutic relief as expected. In this vein, a combinatorial therapy involving drugs having a significant impact on gene and protein expression, and neurological function may be the game changer for FRDA therapy.

Although the clinical trials outcome of DMF has not been published yet, it will be refreshing, if the significant increase in frataxin gene and protein expression reported in preclinical studies will be replicated in FRDA patients (Jasoliya et al., 2019; Sator et al., 2019; Hui et al., 2020). The positive impact of DMF on mitochondrial function reported in preclinical studies further suggests that should this promising drug elicit the desired therapeutic response in FRDA patients; the possibility of administering DMF in combination with omaveloxolone or resveratrol or any of the antioxidants showing a remarkable improvement in neurological function could be an option to explore in future studies. Clinical trials involving the combination of coenzyme Q10 and vitamin E where a slowed neurodegeneration coupled with increased clinical improvements were recorded suggests the possible impact antioxidants could make in FRDA therapy when given as combination therapy (Cooper et al., 2008). Furthermore, combined treatment with deferiprone and idebenone in FRDA indicated a significant reduction in thickness of the interventricular septum (Velasco-Sánchez et al., 2011).

All the antioxidants discussed in this paper were well tolerated and shown to be safe for administration, which is a great step forward. However, there are limitations to these studies. Omaveloxolone itself as the only approved treatment for the disease is very promising, having shown significantly improved mFARS scores in the MOXIe clinical trial. However, it is important to recognise that individuals with clinically significant cardiac disease were excluded from the study (Lynch et al., 2023). Furthermore, there were no echocardiograms or other evaluations of cardiomyopathy carried out in the trial (Lynch et al., 2023) and this is an important factor to consider, especially as cardiomyopathy is the main cause of fatalities in FRDA (Kipps et al., 2009). The study also excluded individuals with *pes cavus* (Lynch et al., 2023), which is one of the main musculoskeletal abnormalities associated with the disease (Milne et al., 2016), and it is unknown what improvements, if any, omaveloxolone would have on FRDA patients with *pes cavus*. Furthermore, whilst combination therapies may be a solution to yielding more effective results in treating FRDA, these must be approached with caution. For example, the clinical trial which investigated the effect of coenzyme Q10 and vitamin E in synergy compared low dose treatments and high dose treatments in FRDA patients without the use of a placebo only group (Cooper et al., 2008). This means that any changes due to placebo effect cannot be identified. Moreover, in the clinical trial evaluating deferiprone and idebenone in combination, the posture and gait scores significantly worsened after 11 months of treatment (Velasco-Sánchez et al., 2011). Overall, the results from these clinical trials indicate that these compounds may affect various tissues in different ways, and thus emphasis should be made on understanding why these effects are seen.

The positive outcomes of clinical trials involving antioxidants to date, pave the way to investigate other antioxidants which have not come into the limelight of FRDA therapy. Although these antioxidants may not show any significant effect on frataxin gene and protein expression preclinically, they may offer some significant improvements on neurological function as we have seen with most of those studied so far. Hence, when an antioxidant can significantly reduce the level of ROS (most especially mitochondria ROS), mitigate oxidative damage, and improve mitochondrial bioenergetics of FRDA mouse models, it justifies further research in this field to comprehensively assess its therapeutic potentials in FRDA.

## Figures and Tables

**Table:** 

**AD**	Alzheimer’s Disease
**ATP**	Adenosine Triphosphate
**ALCAR**	Acetyl-l-Carnitine
**ALS**	Amyotrophic Lateral Sclerosis
**ALT**	Alanine Aminotransferase
**ARE**	Antioxidant Response Element
**AST**	Aspartate Aminotransferase
**DM1**	Myotonic Dystrophy 1
**DMF**	Dimethyl Fumarate
**FARA**	Friedreich’s Ataxia Research Alliance
**FARS**	Friedreich’s Ataxia Rating Scale
**FAST-1**	*FXN* Antisense Transcript 1
**FDA**	Food and Drug Administration
**FRDA**	Friedreich’s Ataxia
**FXN**	Frataxin
**GAA**	Guanine-Adenine-Adenine
**HD**	Huntington’s Disease
**H**_**2**_ **O**_**2**_	Hydrogen peroxide
**hnRNP**	Heterogeneous Nuclear Ribonucleoprotein
**ICARS**	International Cooperative Ataxia Rating Scale
**iPSCs**	Induced Pluripotent Stem Cells
**ISCU**	Iron-Sulfur Cluster Assembly Enzyme
**IRE-BP**	Iron-Responsive Element Binding Protein
**KEAP1**	Kelch-like ECH-Associated Protein 1
**KIKO**	knock-in knock-out
**LOFA**	Late Onset FRDA
**MCRI**	Murdoch Children’s Research Institute
**mFARS**	Modified Friedreich’s Ataxia Rating Scale
**MMF**	Monomethyl Fumarate
**MPP**	Mitochondrial Processing Peptidase
**NADPH**	Nicotinamide Adenine Dinucleotide Phosphate Hydrogen
**NRF2**	Nuclear Factor Erythroid 2-Related Factor 2
**PD**	Parkinson’s Disease
**PMM**	Primary Mitochondrial Myopathy
**PPARγ**	Peroxysome Proliferators-Activated Receptor γ
**PUFA**	Polyunsaturated Fatty Acid
**ROS**	Reactive Oxygen Species
**SARA**	Scale of the Assessment and Rating of Ataxia
**ThDP**	Thiamin Diphosphate
**VLOFA**	Very Late Onset FRDA
